# Combining atomic force microscopy and fluorescence-based techniques to explore mechanical properties of naive and ischemia-affected brain regions in mice

**DOI:** 10.1038/s41598-023-39277-1

**Published:** 2023-08-07

**Authors:** Thomas Fuhs, Bianca Flachmeyer, Martin Krueger, Alexandra Blietz, Wolfgang Härtig, Dominik Michalski

**Affiliations:** 1 Section of Soft Matter Physics, Faculty of Physics and Geosciences, University of Leipzig, Linnéstr. 5, 04103 Leipzig, Germany; 2 Institute for Physical Chemistry, Faculty of Chemistry and Physics, Technical University Freiberg, Leipziger Str. 29, 09599 Freiberg, Germany; 3 Institute of Anatomy, Medical Faculty, University of Leipzig, Liebigstr. 13, 04103 Leipzig, Germany; 4 Department of Neurology, Medical Faculty, University of Leipzig, Liebigstr. 20, 04103 Leipzig, Germany; 5 Paul Flechsig Institute for Brain Research, Medical Faculty, University of Leipzig, Liebigstr. 19, 04103 Leipzig, Germany

**Keywords:** Cellular neuroscience, Diseases of the nervous system

## Abstract

Knowledge of the brain’s structure and function is essential for understanding processes in health and disease. Histochemical and fluorescence-based techniques have proven beneficial in characterizing brain regions and cellular compositions in pre-clinical research. Atomic force microscopy (AFM) has been introduced for mechanical tissue characterization, which may also help investigate pathophysiological aspects in disease-related models such as stroke. While combining AFM and fluorescence-based techniques, this study explored the mechanical properties of naive and ischemic brain regions in mice. Ischemia-affected regions were identified by the green signal of fluorescein isothiocyanate-conjugated albumin. A semi-automated protocol based on a brain atlas allowed regional allocations to the neocortex, striatum, thalamus, hypothalamus, hippocampus, and fiber tracts. Although AFM led to varying measurements, intra-individual analyses indicated a gradually increased tissue stiffness in the neocortex compared to subcortical areas, i.e., the striatum and fiber tracts. Regions affected by ischemia predominantly exhibited an increased tissue stiffness compared to those of the contra-lateral hemisphere, which might be related to cellular swelling. This study indicated intra-individual differences in mechanical properties among naive and ischemia-affected brain regions. The combination of AFM, semi-automated regional allocations, and fluorescence-based techniques thus qualifies for mechanical characterizations of the healthy and disease-affected brain in pre-clinical research.

## Introduction

Along with intensive research on the brain’s structure and function in health and disease, various techniques have been developed to characterize brain regions and different cell types. In animal models, brain sections are widely used for either bright field or immunofluorescence-based histology, allowing, at least in part, simultaneous detection of different cellular and extracellular constituents by applying antibodies, for instance, directed against surface molecules^1–5^. Histochemical techniques were also used to explore, for example, stroke-related consequences to neurons and regionally arranged cellular structures like vessels and the extracellular matrix^6,7^. Insights into the regional arrangement of neurons and associated cellular and extracellular structures have led to the term ‘neurovascular unit’, covering elements affected by focal cerebral ischemia^8–10^. Further development also allowed the visualization of functional consequences such as an impairment of the blood–brain barrier due to focal cerebral ischemia. Thereby, the leakage of substances entering the brain’s parenchyma from the vascular compartment became detectable by histochemical techniques and electron microscopy^6,11,12^.

While continuing efforts in characterizing the healthy and disease-affected brain in more detail, atomic force microscopy (AFM) was introduced as a potential technique to describe mechanical properties. AFM was useful for exploring the properties of neurofilaments as part of the cytoskeleton^13,14^ and vascular elements^15^. Further, AFM has proven beneficial in detecting differences between gray and white matter in slices from the cerebellum of rats^16^. AFM also detected different degrees of stiffness in the hippocampus and the cerebellum, depending on the density of neuronal nuclei and myelin in the brain slices of mice^17^. Recently, AFM was applied to investigate the mechanical properties of hippocampal subregions in brain slices from mice, which showed good accordance with in vivo magnetic resonance elastography^18^. Consistency in measuring mechanical properties by AFM and magnetic resonance elastography was also seen when comparing the hippocampus and the corpus callosum by ex vivo and in vivo investigations in brain slices from mice^19^. Based on these findings in the naive brain, AFM became increasingly attractive for exploring the mechanical properties of brain regions affected by disease-related pathologies. In a pilot study, Michalski et al.^20^ used AFM to investigate parts of rat brains following an experimental stroke and observed a trend toward reduced tissue stiffness due to ischemia. While exploring cytoskeletal changes due to focal cerebral ischemia directly at the ischemic border zone, Mages et al.^21^ regionally correlated measurements of tissue stiffness by AFM in mice. A gradually decreased tissue stiffness in the infarcted area and increased values directly in the ischemic border zone were seen in conjunction with changed intensities of immunohistochemical markers targeting the cytoskeleton.

Taken together, AFM had successfully been used to gain information on the tissue stiffness of hippocampal subregions, the corpus callosum, and the cerebellum in naive brains of mice, which might help to explore cellular and functional specifications. Furthermore, AFM provided information regarding disease-related tissue affections, which might help to understand pathophysiological aspects in more detail. However, a direct comparison of several brain regions has not been performed. Such information might allow a more detailed tissue characterization and estimations of regional sensitivities against disease-related stimuli.

This study aimed to explore the mechanical properties of diverse brain regions, including the cortex, subcortical regions, and tracts of fibers in brain slices from mice by AFM. Moreover, the present work used AFM to explore changes in tissue stiffness in regions of focal cerebral ischemia identified by fluorescence-based techniques.

## Methods

### Study design

In an exploratory approach, this study used brain tissues from mice subjected to unilateral focal cerebral ischemia to address mechanical properties in non- and ischemia-affected brain regions. Analyses were based on four male C57/BL/6J mice with a body weight ranging from 24.4 g to 26.4 g, bred by Charles River Laboratories (Sulzfeld, Germany). Focal cerebral ischemia was applied with a duration of 60 min, as described below. Twenty-four hours after ischemia induction, mice were sacrificed with isoflurane (Isofluran Baxter, Baxter, Unterschleißheim, Germany) and transcardially perfused with saline. One hour before, fluorescein isothiocyanate-conjugated albumin (FITC-albumin) had been injected intravenously to facilitate the identification of infarcted brain areas during further processing^6^. The performed animal experiments followed the European Union Directive 2010/63/EU and the German guideline for the care and use of laboratory animals; reporting followed the ARRIVE criteria^22^. Experiments were approved by the Landesdirektion Sachsen, Leipzig, Germany, as the local authority (reference number TVV 02/17).

### Induction of focal cerebral ischemia

Transient focal cerebral ischemia was conducted by a filament-based model as described originally by Longa et al.^23^ with minor modifications. In brief, followed by a medial cervical skin incision, right-sided cervical arteries were carefully exposed using an operation microscope (Zeiss, Oberkochen, Germany). A silicon-coated 6-0 monofilament (Doccol Corporation, Redlands, CA, USA) was inserted into the internal carotid artery and moved forward until bending was observed or resistance was felt. The filament was removed after a period of 60 min to allow restoration of the blood flow, and the skin was closed with a surgical suture. Surgical procedures were conducted in anesthesia using about 2–2.5% isoflurane (Isofluran Baxter) in a mixture 70% N_2_O/30% O_2_, applied with a vaporizer (VIP 3000, Matrix, New York, USA).

During procedures, cooling was prevented by using a thermostatically controlled warming pad with a target temperature of 37 °C (Fine Science Tools, Heidelberg, Germany). Animals received a complex pain medication including lidocaine (e.g., Licain, DeltaSelect, Dreieich, Germany), meloxicam (e.g., Metacam, Boehringer Ingelheim Vetmedica, Ingelheim, Germany), and metamizol (e.g., Novaminsulfon-ratiopharm, ratiopharm, Ulm, Germany), respectively.

Sufficient induction of focal cerebral ischemia was verified clinically by at least a score of two in the Menzies score^24^, which ranges from zero, indicating no deficit, to four, indicating spontaneous contra-lateral circling, during the observation period after surgery.

### Tissue preparation and measurements on brain tissue’s mechanical properties

Through careful preparation, the brains were removed from the skulls, followed by cutting into slices with a thickness of 350 μm using a vibratome (HM 650 V, ThermoFisher Scientific, Walldorf, Germany). To prevent dehydration, artificial cerebrospinal fluid was applied during the procedure. To allow mechanical characterization by atomic force microscopy, sections were glued onto microscope slides using Histroacryl (Braun, Melsungen, Germany), while one section from each animal was used for final analysis.

For further proceedings, a NanoWizard 4 AFM with 300 μm HybridStage (JPK, Berlin, Germany), added by an Axio Zoom.V16 microscope (Zeiss, Oberkochen, Germany) for fluorescence imaging, was applied. The green fluorescence signal of intravenously injected FITC-albumin was used to identify ischemia-affected brain regions^6,21^, together with other characteristics of focal cerebral ischemia such as tissue damage with consecutive fragmentation or edema formation causing locally restricted or hemispheric swelling. These criteria were also used to identify the contra-lateral, i.e., non-affected hemisphere, which served for measurements in different naive brain regions.

For measurements of tissue stiffness, a contact mode cantilever (Nanoworld, Neuchâtel, Switzerland; spring constant 213 mN/m) was modified with a 6-μm-diameter polystyrene bead to increase the contact area. Force ramps were recorded with the following parameters: maximum force 7.5 nN, z-speed 20 μm/s, z-length 30 μm, 2048 Hz capture rate, and 10 μm data point spacing. During the whole procedure, the coronal brain slices were kept submerged in artificial cerebrospinal fluid to prevent dehydration. AFM data was analyzed with the JPK data processing software (JPK, Berlin, Germany) to calculate the Young’s modulus by fitting a Hertz model to the smoothed force-indentation curves, which were corrected for the respective baseline value.

**Figure 1:**
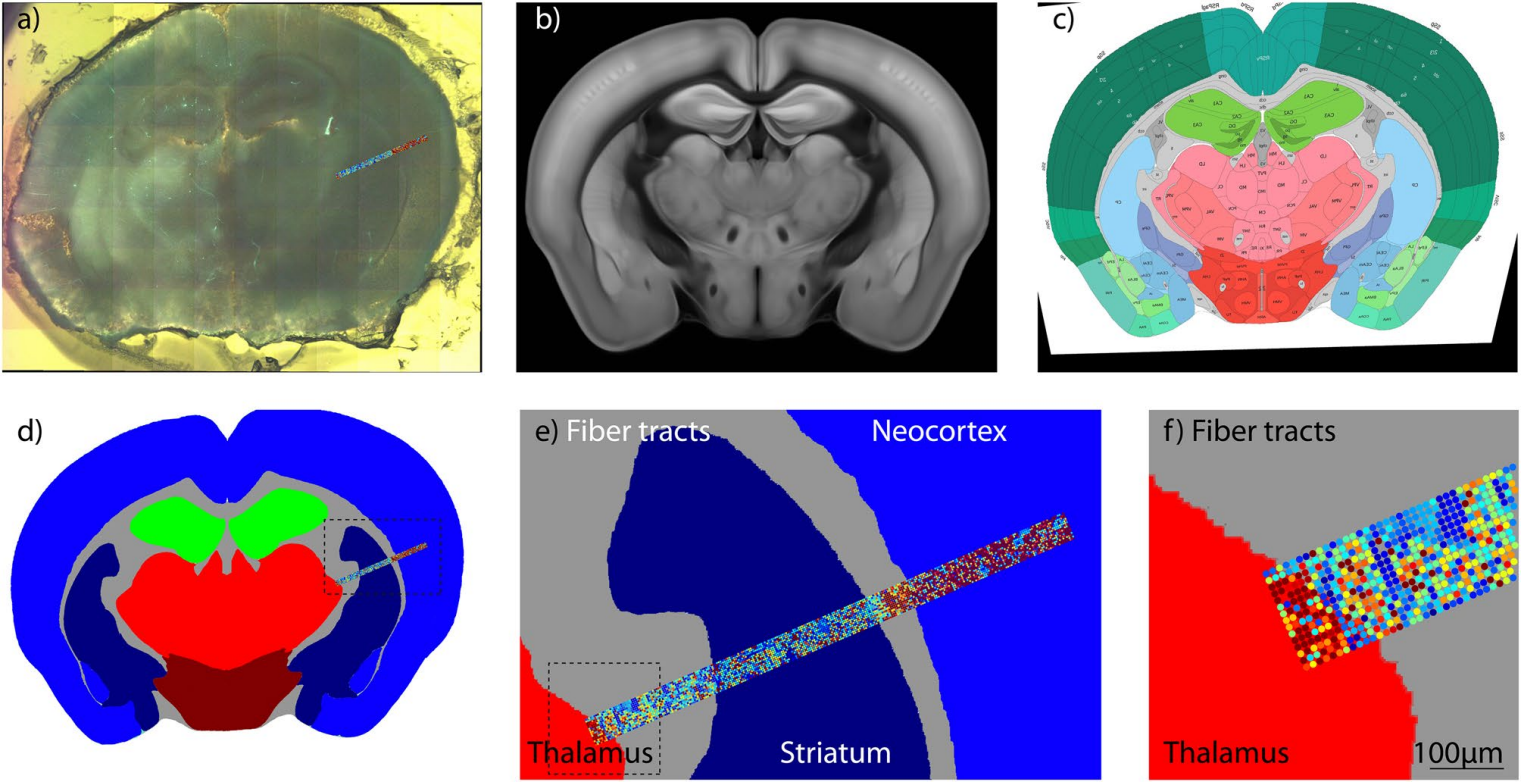
Semi-automatic protocol for an allocation of AFM-based measurements to brain regions based on the Allen mouse brain atlas. Localization of AFM-based measurements (bar with single measurement) overlayed with an image recorded by the optical microscope in the same setup (**a**). The best matching image from the brain atlas (**b**) was registered to the optical image (**c**). The optical image was simplified to 6 regions and AFM-based measurements were grouped accordingly (**d**–**f**).

The AFM image and optical image were recorded in a common reference system using the AFM software calibration routine (Fig. 1a). The Allen Mouse Brain Atlas^25^ was used as the reference for brain regions. From this atlas, a coronal image of the mouse brain was chosen, best matching with the slice applied to measurements of tissue stiffness. The atlas image was then registered to the optical image based on manually selected control points with a custom-written MATLAB (Mathworks, Natick, MA, USA) script (Fig. 1b,c). Next, the atlas image was simplified to 6 regions: Neocortex, striatum, thalamus, hypothalamus, hippocampus, and fiber tracts (Fig. 1d), which allowed the allocation of AFM-based measurements to these brain regions (Fig. 1e,f).

Finally, measurements were grouped by brain regions. Overall, the datasets consisted of about 3000 to 10,000 individual AFM-based measurements per hemisphere distributed over diverse brain regions.

### Statistical analyses

AFM-based measurements were processed with custom MATLAB (Mathworks) scripts for statistical analyses. As distributions showed noticeable deviations from Gaussian distributions, values throughout the text are shown in terms of the median with associated 25th/75th percentiles as Pascal (Pa). Additional data (5th/ 95th percentile, outliers) regarding statistical variance are presented in the figures. The statistical significance of measurements between regions was tested with pairwise Mann–Whitney-U-tests, considering the non-normal distribution of values. Generally, a p-value of < 0.05 was considered statistically significant.

## Results

Four brain sections, each from a different mouse, served for AFM-based measurements of tissue stiffness in diverse brain regions based on Young’s Modulus. For this approach, the non-ischemic hemisphere of each brain section was used. Thereby, the applied semi-automated protocol allowed the allocation of obtained data to the neocortex, striatum, thalamus, hypothalamus, hippocampus, and fiber tracts.

In brain slides from two mice, the region of ischemia was identifiable by an accumulating green fluorescence signal from FITC-albumin, which has been described as a technique for identifying ischemic areas due to impaired blood–brain barrier integrity^6,11,12,21^.

### Mechanical properties of naive brain regions

**Figure 2:**
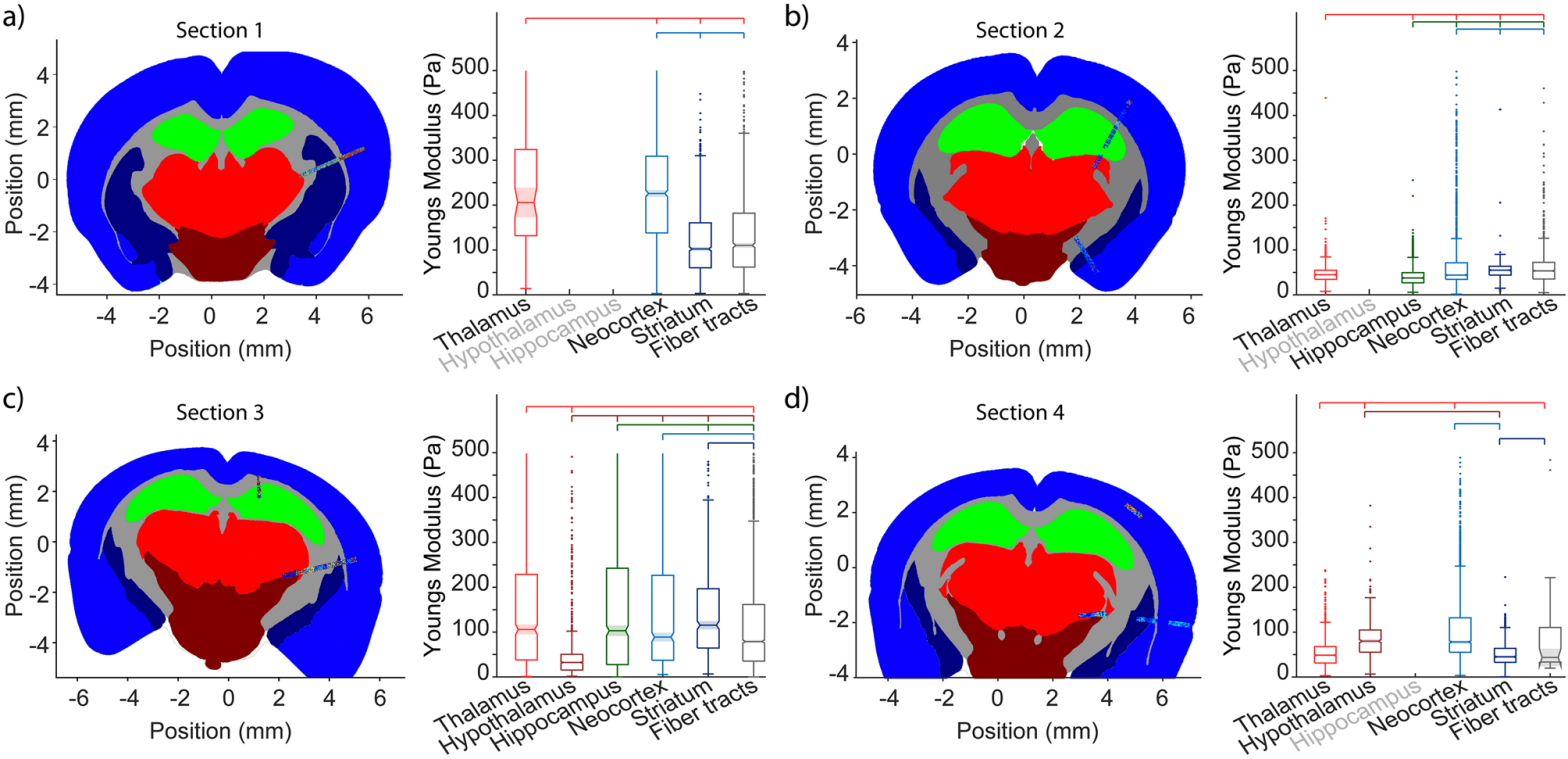
Mechanical properties of naive brain regions. Brain sections of four different mice (**a**–**d**) served for AFM-based measurements, while the originating data was grouped according to brain regions. On the intra-individual level, significant differences in tissue stiffness were found for most regions. Displayed plots indicate the median, the 1st and 3rd quartile, whiskers indicate 5th and 95th percentiles and the notch the variance of measurements. Significant differences at the p < 0.05 level are indicated by added lines, starting with the left group with ticks for all other groups that are different.

In the first brain section analyzed, AFM-based measurements were allocated to the neocortex (224.6, 135.9/307.9 Pa), striatum (100.0, 57.7/158.7 Pa), fiber tracts (108.1, 59.2/180.2 Pa), and thalamus (204.3, 129.8/323.0 Pa), whereby the latter was characterized by only a few measuring points (Fig. 2a). Although a high variation of values was seen, tissue stiffness was significantly increased in the thalamus and the neocortex compared to the striatum and fiber tracts (all p < 0.05). Further, tissue stiffness of the neocortex was slightly increased as compared directly with the thalamus (p < 0.05). However, the tissue stiffness did not differ significantly between the striatum *and* fiber tracts.

In the second brain section analyzed, AFM-based measurements were allocated to the thalamus (43.4, 33.0/53.4 Pa), hippocampus (36.2, 25.3/48.1 Pa), neocortex (42.6, 33.0/70.1 Pa), striatum (53.6, 42.6/62.4 Pa), and fiber tracts (52.0, 33.8/71.3 Pa) (Fig. 2b). Overall, tissue stiffness appeared lower than in the first brain section, while high variations of values were detected again. However, a significantly decreased tissue stiffness was found in the hippocampus compared to the thalamus, the neocortex, the striatum, and fiber tracts. Further, tissue stiffness of the thalamus was slightly increased compared to the hippocampus and lower than in fiber tracts (both p < 0.05). However, the tissue stiffness of the cortex, striatum, and fiber tracts did not differ significantly.

AFM-based measurements in the third brain section investigated included the thalamus (106.1, 37.9/229.5 Pa), hypothalamus (32.5, 15.5/50.5 Pa), hippocampus (103.4, 27.7/243.3 Pa), neocortex (89.1, 37.3/227.4 Pa), striatum (115.8, 64.7/197.3 Pa), and fiber tracts (79.2, 35.4/162.1 Pa) (Fig. 2c). Although high variations were observed again, tissue stiffness of the hypothalamus was significantly decreased compared to all other regions (all p < 0.05). Further, the tissue stiffness of fiber tracts was decreased with reference to the striatum, neocortex, thalamus, and hippocampus (all p < 0.05). However, the neocortex and striatum did not differ significantly regarding tissue stiffness, although gradually higher values were recorded in the neocortex.

In the fourth brain section analyzed, AFM-based measurements covered the thalamus (48.7, 31.1/68.0 Pa), hypothalamus (80.2, 55.5/105.1 Pa), neocortex (78.3, 55.2/132.4 Pa), striatum (45.2, 32.7/64.1 Pa), and fiber tracts (43.8, 33.4/110.8 Pa), whereby a high variation of values was in particular seen in the neocortex (Fig. 2d). Tissue stiffness was significantly decreased in the thalamus compared to the hypothalamus, neocortex, and fiber tracts (all p < 0.05). Further, tissue stiffness of the hypothalamus was increased with reference to the striatum (p < 0.05). The numerically most increased tissue stiffness was found for the neocortex with a statistically significant difference also with respect to the striatum (p < 0.05).

### Mechanical properties of brain regions affected by focal cerebral ischemia

**Figure 3:**
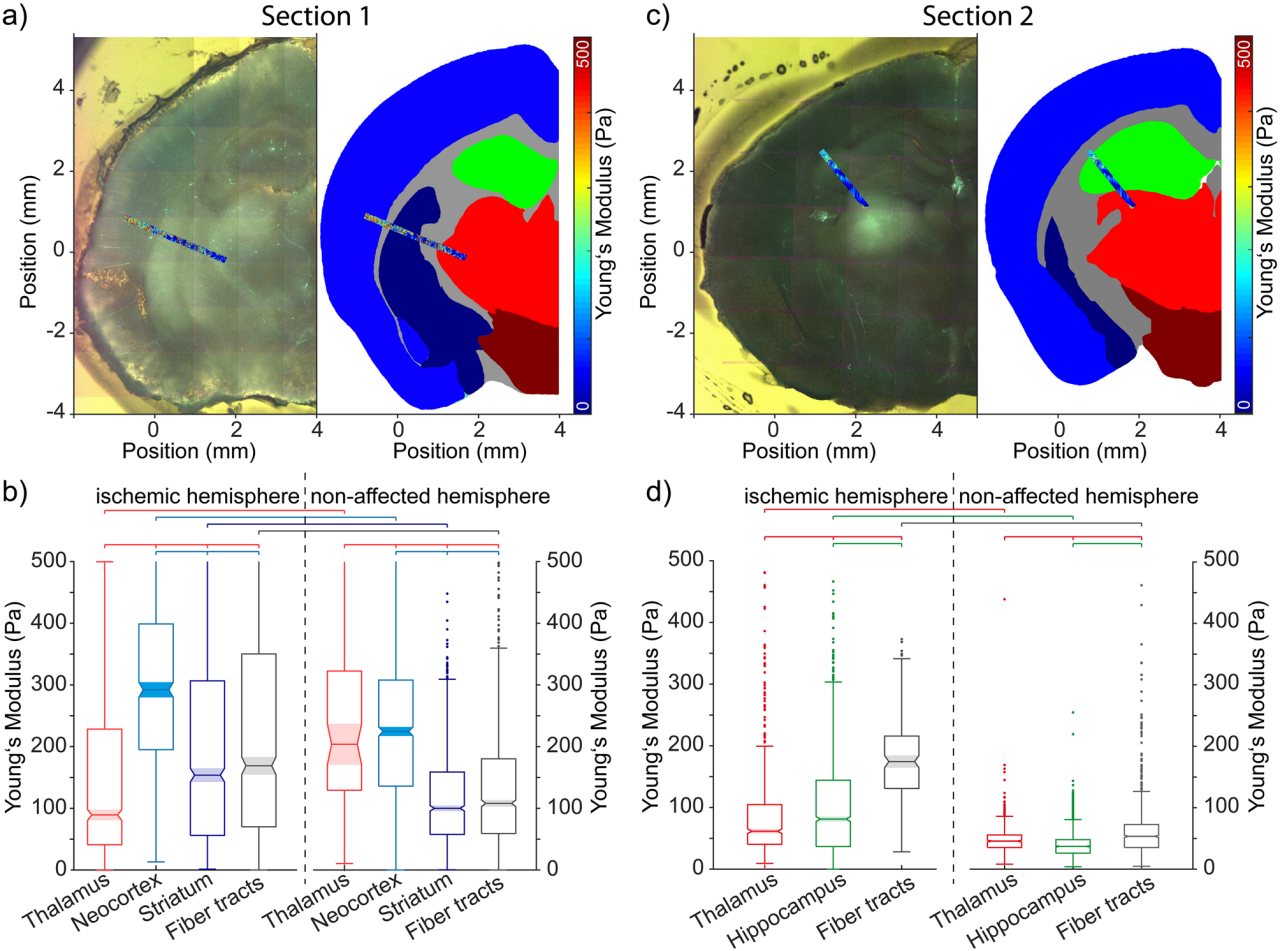
Mechanical properties of brain regions affected by focal cerebral ischemia. In brain sections of two different mice, an accumulating green fluorescence signal of FITC-albumin indicated the area of focal cerebral ischemia. AFM-based measurements covering the ischemic and neighboring areas were allocated to brain regions. Displayed plots indicate the median, the 1st and 3rd quartile, whiskers indicate 5th and 95th percentiles and the notch the variance of measurements. Significant differences at the p < 0.05 level are indicated by added lines, starting with the left group with ticks for all other groups that are different.

The ischemic area in the first brain section comprised both the cortex and subcortical regions (Fig. 3a,b). AFM-based measurements were allocated to the neocortex (292.1, 195.0/398.9 Pa), striatum (153.7, 56.1/306.5 Pa), thalamus (89.5, 41.3/228.2 Pa), and fiber tracts (167.7, 69.5/346.4 Pa). Comparing the ischemia- with the non-affected hemisphere, tissue stiffness was significantly increased due to ischemia in the neocortex, striatum, and fiber tracts (all p < 0.05). Tissue stiffness in the ischemia-affected thalamus was decreased with reference to the contra-lateral hemisphere (p < 0.05), whereby the relatively small number of measuring points of the non-affected hemisphere needs to be considered. Further, while comparing brain regions within the ischemia-affected hemisphere, tissue stiffness of the neocortex was significantly increased with reference to the striatum and fiber tracts (all p < 0.05). In contrast, the stiffness of the thalamus was significantly decreased compared to the other regions (all p < 0.05).

The ischemic alterations in the second brain section analyzed mainly affected subcortical areas (Fig. 3c,d). AFM-based measurements were allocated to the thalamus (61.6, 40.4/104.7 Pa), hippocampus (81.3, 36.7/144.2 Pa), and fiber tracts (174.4, 130.8/215.8 Pa). When comparing the ischemia- with the non-affected hemisphere, tissue stiffness was increased in the ischemic thalamus, hippocampus, and fiber tracts (all p < 0.05). Within the ischemia-affected hemisphere, tissue stiffness of the thalamus was significantly reduced with reference to the hippocampus and fiber tracts (both p < 0.05). Further, tissue stiffness of the hippocampus was significantly decreased compared to fiber tracts (p < 0.05).

## Discussion

This study was aimed to explore the mechanical properties of diverse brain regions in mice applying AFM as a technique that might allow a more detailed tissue characterization under naive and disease-related conditions. As one of the main findings, measuring tissue stiffness in brain sections from mice followed by allocation to different brain regions appears feasible when combining AFM and a semi-automated protocol that refers to data from a brain atlas^25^. Furthermore, adding a fluorescence-based technique enabled AFM-based measurements in areas of focal cerebral ischemia.

While applying AFM in brain sections from different mice, a high variation of measurements was seen among the sections investigated and, thus, among mice. Considering the variety of possible reasons, this observation is likely related to the complex experimental setup with a need for AFM calibration at the level of each section, although equal parameters were used for measuring Young’s Modulus. However, on an intra-individual level, AFM-based measurements allowed good comparison of tissue stiffness between diverse brain regions, i.e., the neocortex, striatum, thalamus, hypothalamus, hippocampus, and fiber tracts.

As an observation that was consistently made in all four brain sections investigated, tissue stiffness of the neocortex appeared gradually increased when compared to the striatum and fiber tracts. Fiber tracts were thus not identified as the region with the highest stiffness, which could have been expected when considering the natural density of fibers in this area. With reference to earlier investigations on tissue stiffness in the naive brain, Christ et al.^16^ reported on AFM-based measurements in the cerebellum of rats and found that the gray matter, i.e., the cerebellar cortex, was approximately 50% stiffer than the regionally associated white matter. Regarding subcortical regions, the present study revealed a relatively increased tissue stiffness of the thalamus compared to the other brain regions in two sections. In contrast, the two other sections indicated a rather decreased stiffness in the thalamus. Further, the hippocampus was in one section measured with relatively decreased stiffness and in another section with enhanced tissue stiffness. These variations might be explained by the complex composition of the hippocampus with several layers and different cellular densities^26^, which may lead to different degrees of stiffness depending on the layer investigated. The difficulties of measuring tissue stiffness in hippocampal areas are highlighted by Bertalan et al.^19^: They focused on the hippocampus and corpus callosum while using AFM in brain sections from mice (ex vivo) and magnetic resonance elastography in mouse brain sections and the vital human brain (ex vivo and in vivo) with, in the end, inverse findings for the hippocampus. The assumption of significant regional differences within hippocampal structures is further supported by a recent study from Morr et al.^18^, who used AFM for regional measurements in brain sections from mice and found a significantly decreased tissue stiffness in the subgranular zone compared to the surrounding granular cell layer and molecular layer of the dentate gyrus. Addressing the stiffness of cerebral subregions or even regionally associated cells is generally hampered by the challenging identification, as AFM, in its single use, does not allow specific allocation. Nevertheless, a combined approach of fluorescence-based techniques and AFM, also requiring the combination of fluorescence microscopy for visualization and AFM for mechanical characterization appears as promising strategy. Concerning the fluorescence signal used for visualization, it seems feasible to use genetically altered mice as done in the study by Morr et al.^18^, in which a green fluorescence protein is expressed under the nestin promoter. Alternatively, externally applied substances were shown to be useful in the present work and earlier studies^20,21^, where FITC-Albumin was intravenously injected with consecutive extravasation in ischemic brain areas.

Applying a combined technique for fluorescence-based visualization of the ischemic area and AFM-based measurements of tissue stiffness, this study indicated an increased stiffness in the ischemia-affected neocortex, striatum, hippocampus, fiber tracts, and at least in part the thalamus, compared to the contra-lateral, i.e., non-affected, hemisphere. Thereby, the finding of increased tissue stiffness in ischemic areas supports data from Mages et al.^21^, who investigated an ischemic lesion that reached the neocortex and observed a gradually increased stiffness directly in the ischemic border zone. However, Mages et al.^21^ also observed a gradually decreased tissue stiffness in the ischemic area more distant from the border zone and thus more severely affected by the ischemic stimulus. Although observations of the present study and those made by Mages et al.^21^ are based on mice, they could be seen in line with findings from an earlier study from Michalski et al.^20^: Investigating brain sections from rats, a gradually decreased stiffness in the ischemic subcortical region was found, likely representing the most severely affected region, compared to the contra-lateral hemisphere and the ipsi-lateral border zone of ischemia. Even though cumulative evidence for an increased tissue stiffness due to focal cerebral ischemia emerged from the present and earlier investigations^20,21^, its interpretation remains challenging. Among the pathophysiological consequences of focal cerebral ischemia^27,28^, (i) cellular swelling due to water influx, (ii) different degrees of affection according to the penumbra concept, which includes a shell-like configuration with maximum damage in the center, and (iii) time-dependent effects with shifting mechanisms of tissue damage over time appear relevant. An increased tissue stiffness, which seems to occur predominantly within the ischemic border zone, might thus be related to a local accumulation of swelling cells exhibiting signs of ischemia but not yet a disruption of cell membranes. This interpretation is supported by an earlier study in mice, describing cellular edema in conjunction with a decreased density of cell-stabilizing elements as seen by electron microscopy in areas of focal cerebral ischemia^21^. However, in areas with more severe ischemic affection, cellular degeneration may ultimately result in membrane disruption and, thus, a loss of cellular integrity, whose mechanical correlate might be a decreased tissue stiffness.

The present study has a few methodological limitations: The number of animals is relatively small, thus preventing the generalization of the findings. While selected images from the mouse brain atlas^25^ strictly followed a coronal orientation, obtained brain sections might, at least in part, slightly differ from this direction due to challenges when cutting non-fixed brain tissue, which may hamper regional allocations across the overall section. A digital, three-dimensional brain atlas that can extract images with respective orientations might help to overcome this issue. Further, FITC-albumin was used to identify the area of focal cerebral ischemic, as this technique has proven applicable when resulting in an apparent regional accumulation within the territory of the ischemia-affected middle cerebral artery^6^. However, in two out of four brain sections included in this study, identifying the ischemic area by an accumulating green signal was difficult, e.g., by an atypical appearance of the signal, which prevented allocation and thus measurements of ischemic regions in these cases. Therefore, future studies are needed to refine techniques for identifying brain regions affected by disease-related models, which in parallel allow AFM-based measurements. In addition to brain tissues originating from animals, those derived from humans might be of special interest to verify disease-related alterations of mechanical properties seen in pre-clinical work. This ambitious goal should result in standardized protocols, including the entire process from tissue preparation and potential fixation^29^ to measurements in distinct brain regions.

Despite some limitations, the present study indicated intra-individual differences in mechanical properties among naive and ischemia-affected brain regions in mice while combining AFM, a semi-automated protocol for regional allocations, and fluorescence-based techniques. Combined approaches like this one might be helpful for pre-clinical research by facilitating a more detailed characterization of the healthy and disease-affected brain. Changed mechanical properties might also represent additional criteria when exploring the effects of pharmacological and other interventions in pre-clinical studies.

## Data Availability

Data underlying this study will be made available by the corresponding author upon reasonable request.
